# Metastatic Lung Cancer to the Head and Neck: A Clinico-Pathological Study on 21 Cases with Narrative Review of the Literature

**DOI:** 10.3390/jcm12041429

**Published:** 2023-02-10

**Authors:** Saverio Capodiferro, Antonio d’Amati, Giuseppe Barile, Fabio Dell’Olio, Luisa Limongelli, Angela Tempesta, Rosaria Arianna Siciliani, Giuseppe Ingravallo, Mauro Mastropasqua, Giuseppe Colella, Ciro Emiliano Boschetti, Chiara Copelli, Eugenio Maiorano, Gianfranco Favia

**Affiliations:** 1 Unit of Odontostomatology, Department of Interdisciplinary Medicine, University of Bari “Aldo Moro”, 70124 Bari, Italy; 2 Unit of Pathological Anatomy, Department of Department of Precision and Regenerative Medicine and Ionian Area, University of Bari “Aldo Moro”, 70124 Bari, Italy; 3 Unit of Maxillo-Facial Surgery, Multidisciplinary Department of Medical, Surgical and Dental Speciality, University of Campania “Luigi Vanvitelli”, 80138 Napoli, Italy; 4 Unit of Maxillo-facial Surgery, Department of Interdisciplinary Medicine, University of Bari “Aldo Moro”, 70124 Bari, Italy

**Keywords:** lung cancer, metastatic lung cancer, oral metastasis, head and neck metastasis, oral cavity, immunohistochemistry

## Abstract

Metastases from lung cancer to the oral cavity and to the head and neck generally are very infrequent and usually manifest in advanced stages of the disease. Even more rarely, they are the first sign of an unknown metastatic disease. Nevertheless, their occurrence always represents a challenging situation both for clinicians, in the management of very unusual lesions, and for pathologists, in the recognition of the primary site. We retrospectively studied 21 cases of metastases to the head and neck from lung cancer (sixteen males and five females, age range 43–80 years; eight cases localized to the gingiva [two of these to the peri-implant gingiva], seven to the sub-mandibular lymph nodes, two to the mandible, three to the tongue, one case to the parotid gland; in eight patients, metastasis was the first clinical manifestation of an occult lung cancer) and proposed a wide immunohistochemical panel for a proper identification of the primary tumor histotype, including CK5/6, CK8/18, CK7, CK20, p40, p63, TTF-1, CDX2, Chromogranin A, Synaptophysin, GATA-3, Estrogen Receptors, PAX8, PSA. Furthermore, we collected data from previously published studies and narratively reviewed the relevant literature.

## 1. Introduction

Metastatic tumors to the head and neck are rare and usually occur in the advanced stage of malignancy [1,2,3,4,5,6,7]. Their incidence is between 1% and 8% considering oral malignant tumors and, with the exception of childhood malignant neoplasms, the peak incidence is in the fifth to seventh decades [2,3,6,7]. Occasionally, they represent the first clinical sign of an occult cancer or, more frequently, they can be discovered during clinical follow-up of patients with known primary carcinomas [1,2,3,4,5,6,7].

Metastases to the oro-facial tissues may involve the oral mucosa (mostly the gingiva and more rarely the tongue, with a prevalence of 0.17%), the jawbones (especially the mandible), the salivary glands, the soft tissues and the post-extraction sites. The most common primary site may differ on the basis of gender; in males, the most frequent origin of oro-facial metastases is the lung, followed by the kidney, prostate and colon-rectum, while in females it is the uterus, followed by the breast, lung and ovary [4,5,8,9,10,11,12]. Adenocarcinoma is the most common histologic type of primary lung cancer (LC), which is furthermore showing an increasing incidence worldwide [13]. LC most frequently metastasizes to regional lymph nodes, but hematogenous metastases to extra-pulmonary organs such as the brain, bones, liver and adrenal glands may commonly occur [14]. Metastases to the head and neck are very exceptional, representing only approximately 3% of LC metastatic sites, though, in a limited number of reports, they have also been described as a possible initial manifestation of the disease [15,16,17]. In these cases, the oral localization of an occult LC may surely represent a diagnostic challenge, especially when the clinical work-up remains restricted to the cervico-facial region.

Herein, we report on the clinical-pathological features and the multidisciplinary diagnostic work-up of 21 patients with head and neck metastasis from lung carcinoma, placing more emphasis on those cases in which the head and neck metastasis represented the first manifestation of the disease. Moreover, we reviewed the relevant literature on metastatic lung cancer of the head and neck.

## 2. Materials and Methods

The clinical charts of patients with secondary neoplasms occurring in the head and neck and diagnosed at the Interdisciplinary Department of Medicine -Unit of Odontostomatology- of the University of Bari “Aldo Moro” and at the Unit of Maxillo-Facial Surgery- University of Campania “Luigi Vanvitelli” during the period 1990–2020 were collected. Among these, 21 cases of metastatic LCs with histopathological evaluation of both the primary and the metastatic localizations were selected and included in this study. Data regarding age, sex, primary tumor, clinical presentation, and any additional relevant information, when available, were collected.

Lesions localized at the parotid gland and the submandibular lymph nodes underwent fine needle aspiration, both for cytological (FNAC) and histological examination (FNAB). In all other cases, a surgical sample was collected, consisting of an excisional biopsy, whenever possible; this procedure was particularly indicated for the intra-oral lesions also needed to improve chewing and speaking functions in several cases with large lesions.

The surgical samples had been formalin-fixed, paraffin-embedded and stained with hematoxylin-eosin (H&E). Based on the morphological features of the tumors, which did not fit with conventional mucosal, odontogenic or salivary gland carcinomas, special stains and immunohistochemical investigations were employed in all cases and, for this purpose, additional sections were cut from the paraffin blocks and stained with mucicarmine, periodic acid-Schiff (PAS), with and without diastase treatment, and with immunohistochemical stains, which were carried out with a modified avidin-biotin peroxidase technique, using an automated immunostainer (Autostainer, Agilent Technologies, Glostrup, Denmark); the primary antibodies included CK5/6, CK8/18, CK7, CK20, p40, p63, TTF-1, CDX2, Chromogranin A, Synaptophysin, GATA-3, Estrogen Receptors, PAX8, PSA, as listed in Table 1.

This study was carried out in accordance with the code of ethics of the World Medical Association (Declaration of Helsinki) and approved by the internal ethical committee (study number 4575, prot. 1442/C.e). Patients released informed consent on diagnostic and therapeutic procedures and for the possible use of biological samples for research purposes.

## 3. Results

Clinical data are summarized in Table 1. The patient group was composed of sixteen males and five females, and the age range was from 43 to 80 years old. In eight cases the metastases involved the gingiva (two of these cases were localized to the peri-implant gingiva; such cases have been already published) [8]; in seven cases the sub-mandibular lymph nodes, in two cases the mandible (intra-osseous), in three other cases the tongue, and in one case the parotid gland. In eight patients, the metastasis was the first clinical manifestation of an occult LC, while in the remaining cases it was an adjunctive localization of a multi-organ metastatic disease.

Metastases to the parotid gland and submandibular lymph nodes showed the classic appearance of rapidly progressing and painless swellings, and the diagnosis was achieved by histological examination of cytological or biopsy samples obtained either by FNAC or FNAB, respectively. Surgical biopsies were obtained from patients with lesions localized in all the remaining sites.

**Figure 1:**
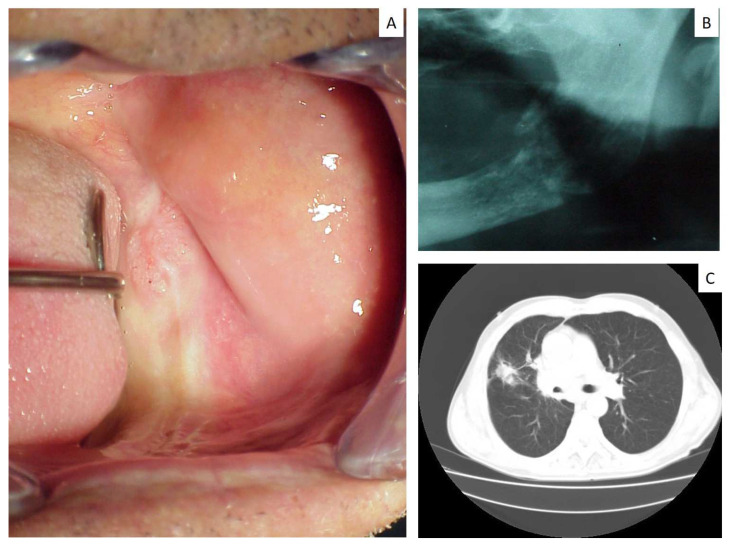
Swelling of the retromolar trigone covered by normally colored mucosa (**A**) with infiltration of the underlining bone (**B**), diagnosed as metastasis from LC (**C**).

**Figure 2:**
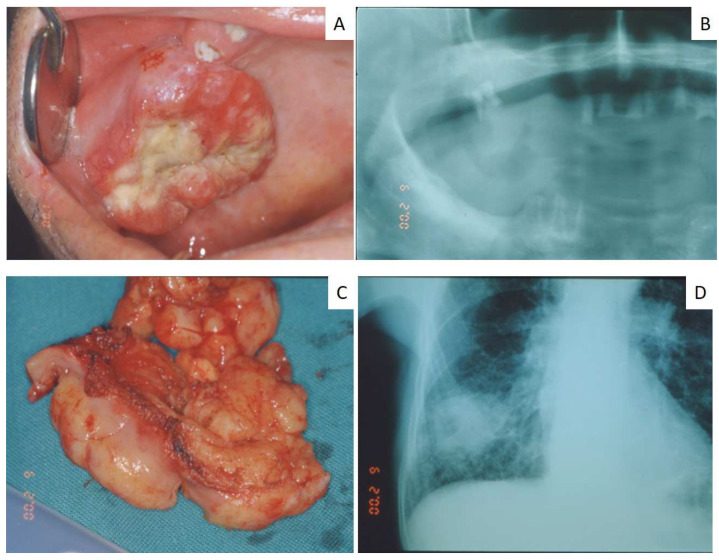
Wide ulcerated lesion of the adherent maxillary gingiva extended to the palate and the cheek mucosa (**A**), associated with a residual decayed dental root (**B**); the lesion was removed (**C**) to improve function and finally resulted as the first manifestation of an unknown non-small cell lung carcinoma (**D**).

Almost all the gingival metastases (both in the mandible and maxilla) showed the clinical appearance of a rapidly growing lesion, with a reddish color and interspersed yellowish areas (probably related to the biting trauma), (Figure 1 and Figure 2); these lesions were usually painful and associated with poor dental condition and gingival inflammation (decayed teeth, necrotic dental roots, etc.). Larger gingival metastases were responsible for speaking and chewing difficulties.

**Figure 3:**
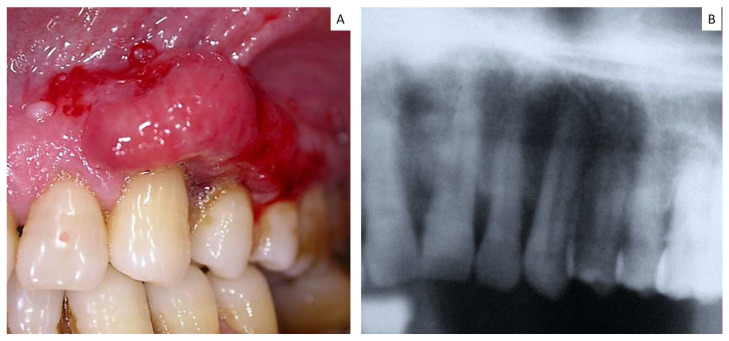
Gingival reddish enlargement involving the 3.2, 3.3 and 3.4 (**A**) with alveolar bone involvement, (**B**) mimicking a periodontal lesion but unresponsive to the conventional procedure of dental hygiene and full mouth disinfection, thus needing a biopsy sampling; the histological diagnosis revealed it as the first manifestation of an unknown adenocarcinoma of pulmonary origin.

**Figure 4:**
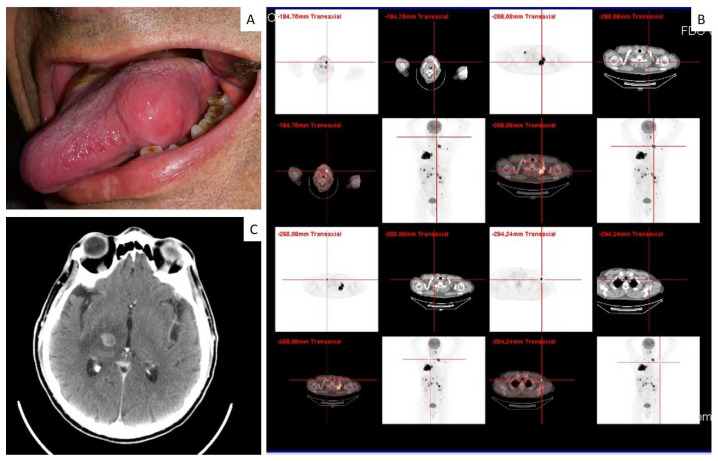
Metastatic lesion of the tongue (**A**) occurring in a patient already diagnosed with small cell carcinoma of the lung, as evident in PET scan (**B**), and with simultaneous brain metastasis (**C**).

**Figure 5:**
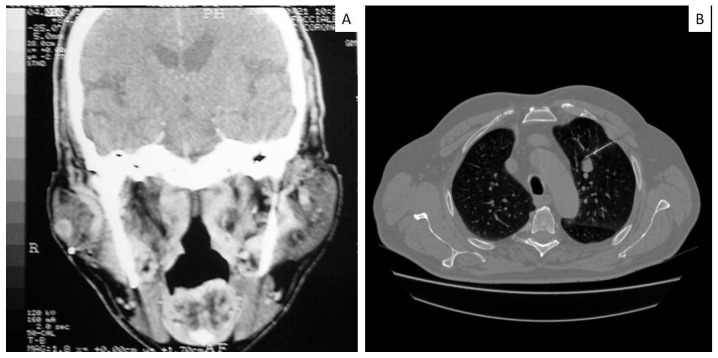
Non−specific parotid enlargement (**A**) occurring in a patient with an unknown small cell lung carcinoma, subsequently detected during the clinical work−up for the primitive tumor identification (**B**).

In one case, the gingival lesion occurred on the adherent gingiva of upper premolars, showing a relatively small size and thus resembling a common periodontal disease also at the periapical radiograms (Figure 3). Inflammatory signs, such as perimplantisis and/or perimucositis, were also detected in two cases located around the dental implants (such cases have been previously published) [8]. Tongue metastases clinically appeared as submucosal nodular masses (Figure 4), while parotid gland involvement manifested as a non-specific swelling (Figure 5).

In the eight cases with head and neck metastases presenting as the first clinical sign of occult LC, the chest radiogram and/or computed tomography confirmed the presence of a still undetected lung nodule, compatible with primary LC and subsequently confirmed by a histological examination.

**Figure 6:**
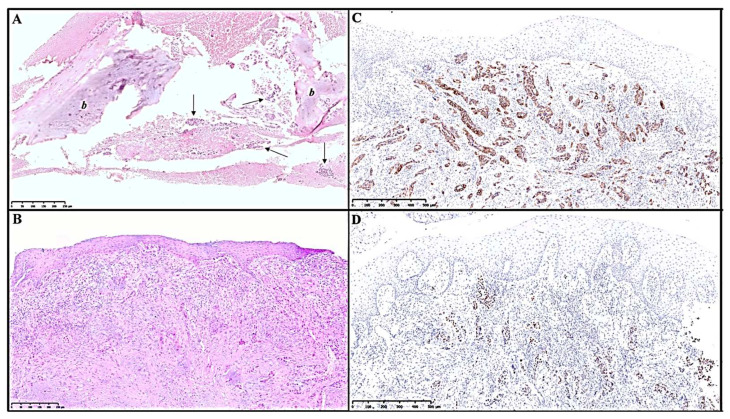
(**A**) Histological image of an intraosseous metastasis from lung adenocarcinoma, showing bone fragments (*b*) intermingled with groups of neoplastic cells (arrows) [100×, H.E.]; (**B**) Oral submucosal infiltration of malignant poorly formed glands [100×, H.E.]; (**C**) Immunohistochemical staining showing CK7 positivity in neoplastic glands [50×, H.E.]; (**D**) Immunohistochemistry reveals positivity for TTF-1 in neoplastic cells, suggesting lung origin [50×, H.E.].

Overall, head and neck metastases were first diagnosed by histological examination, revealing intraosseous (Figure 6A) and/or submucosal (Figure 6B) localization of carcinomas, and were successively confirmed by immunohistochemical staining, in order to assess the tumor type and origin. Adenocarcinomas showed a strong positivity for CK7 (Figure 6C) and TTF−1 (Figure 6D); the enteric-type variant also showed focal immunoreactivity for CDX2 and CK20; small cell carcinomas were positive for Synaptophysin, Chromogranin A, CK8/18 and TTF-1, with a very high Ki67 proliferative index; squamous cell carcinomas were positive for CK 5/6, p63 and p40; undifferentiated carcinomas (non-small cell carcinoma—unclassifiable) were positive for CK AE1/AE3, focally for TTF-1, and occasionally stained for CK7, CK 5/6 and p63. All of them were negative for estrogen receptors, GATA-3, PAX8 and PSA (Table 2).

## 4. Discussion

### 4.1. Lung Cancer

#### Epidemiological Data

Lung cancer is the most common malignancy worldwide, with 1.76 million deaths per year, thus representing one of the major public health problems [16]. As far as the epidemiology is concerned, in Western countries and in most of Asian countries, among all histologic variants of LC, adenocarcinoma has overtaken squamous cell carcinoma, previously representing the most frequent type. According to the data reported by the Surveillance, Epidemiology and End Results cancer statistics (period 2011–2015 among the US population), 47.9% of LCs are histologically adenocarcinoma, 23.2% squamous cell carcinoma, 12.9% small cell carcinoma, and 15.9% other subtypes [11,17].

### 4.2. Lung Cancer Metastasis

#### 4.2.1. Establishment, Invasion and Cell Migration

In common with other malignancies, the neoplastic transformation in LC is followed by tumor growth supported by vascular supply, stroma invasion by escaping the immune cell attack and, finally, migration (and epithelial to mesenchymal transition). These steps occur by a not fully understood mechanism, that are essentially driven by the escaping of apoptosis and cell death and induced in hypoxic areas of the proliferating tumor [18,19]. The following steps of the invasion–metastasis cascade are the blood/lymphatic vessel invasion and the survival as circulating tumor cells. Blood vessel invasion occurs by the formation of holes in the matrix and vessel basal lamina by protein degradation and migration into the intima [15,20]. The invasion of the thinner walls of lymphatic vessels occurs in an easier way, compared to the blood vessels. The survival of circulating tumor cells is related to their capability to overcome the shear stress and to escape the immune system [4,15,21]. In fact, most of the tumor cells do not survive within the circulation when they express the remaining cytokeratin filaments, while those expressing increased or de-novo α-actin and Vimentin may easily adapt to small vessels diameters [15]. Moreover, the recruitment of monocytes/macrophages by tissue factor-mediated coagulation is essential for cell survival and promotes cell spreading by extravasation [18,22]. Generally, the hematogenous diffusion promotes an earlier onset of distant metastasis from LC, compared to the lymphogenous pathway. Migration can occur by single cells or small clusters (mostly in small-cell and undifferentiated carcinomas), which are probably more effective thanks to higher cell adaptability, or by large clusters of organized cells (mostly in acinar adenocarcinoma and rarely in squamous cell carcinoma) [4,15,20].

#### 4.2.2. Metastatic Colonization, Site Specificity, and the Role of Inflammation at the Intraoral Metastatic Sites

The “metastatic cascade” is a highly inefficient process, as several factors promote cell migration and epithelial-mesenchymal transition but a very low number of tumor cells survive within the circulating system and effectively reach the target organ; additionally, the latter must provide a favorable microenvironment for metastatic colonization [15,23]. Nevertheless, most LCs are diagnosed in advanced stages; hence, most patients die because of metastases, particularly to the brain and bones [16,23,24,25].

In fact, the brain is the most frequent metastatic site of LC, frequently showing diffuse infiltration by small cell carcinoma or nodular subcortical formations by adenocarcinoma [20]. Pulmonary metastases are also very frequent in LC patients and additionally via a poorly defined mechanism [20,23,24,25,26,27,28,29,30]. The bone metastases show a quite peculiar process, as cells should interact with a different stroma and bone marrow. Such a mechanism has been relatively clarified in different studies, aimed at improving therapy outcomes (e.g., PDGFRβ inhibition leading to growth inhibition and apoptosis induction, or knockdown of discoidin domain receptor 1 by siRNA reducing invasiveness into collagen matrices by decreased osteoclast activity and increasing apoptosis, inhibition of TGF-β and metalloproteinases and reducing bone tumor burden, RHOB silenced by siRNA inhibiting bone metastasizing in mice, fully humanized monoclonal antibody against RANKL for prevention and delay of bone metastatic lesions) [31,32,33,34,35,36,37,38]. Nevertheless, it is still hard to fully understand the different metastatic steps (preparation of the niche, cell homing, interaction with the stroma, access to blood vessels and finally growth regulation at the metastatic site) and to find new treatments capable of counteracting this process [15,20,30].

Distant bone involvement in lung cancer cells generally manifests as osteolytic lesions, additionally in the jaw (mainly in the posterior mandible and mostly originating from adenocarcinoma). The gingiva is also reported to be frequently involved in metastases [2,3,4,5,6,7,39,40,41]. With regard to this, following the analysis of numerous studies in the literature, there is an evident need to distinguish lesions involving the gingiva or the bone (intraosseous) alone, from those involving both. Additionally, in some cases it is difficult to assess whether the true origin is from soft (gingiva) or hard (bone) tissue, especially in the presence of teeth or dental implants. The definition of periodontal metastases could probably fit better in such cases. Moreover, according to several studies, an inflammatory microenvironment at the distant site may surely represent a favourable pre-metastatic niche for implantation, proliferation and further dissemination [42,43,44]. The possibility of detecting an inflammatory condition in the oral cavity, especially of the gingiva-periodontal tissues, is relatively high. Furthermore, the presence of teeth and/or dental implants has been reported to be strongly associated with malignancy occurrence, including metastatic onset and especially in an advanced metastatic disease; such associations may be related to the presence of periodontal/peri-implant inflammation [37,38,45,46,47,48].

#### 4.2.3. Relevant Literature

It is generally accepted that the oral region is not a preferred site for metastatic colonization, especially in cases of LC [36,37]. There are several studies regarding this, mostly representing single cases or small case series. Occurrence in the jawbone is generally reported in the advanced stages (III and IV) of LC (usually with multiorgan involvement); hence, many lesions long remain undiagnosed in such patients, especially when clinical symptoms (mostly pain, swelling, bleeding) and functional limitations in chewing and/or speaking are not significant. Therefore, the true incidence of metastases in the oral cavity is probably underestimated. Of note are surely the numerous studies of the study group of Hirshberg A. [1,3,4,6,12,36,49,50,51,52], which represented from 1993 onwards a solid reference on such topics both for epidemiologic and clinic-pathological data. Their largest review of 2008 studied 673 cases [36] of metastatic tumors in the oral cavity, thus grouping both jawbones and soft tissue. The authors stated that, overall, the lung was the most common primary site (112 cases) and that LC metastases affected both jawbones (mostly the mandible) and oral mucosa (58 and 54 cases, respectively). An extensive review of the literature was performed in 2016 and updated in 2017 by Irani S. [7], focusing exclusively on metastasis to the jawbones and grouping 453 different cases. Sixty-six were metastatic lesions from LC (mostly in men—49 cases — and mainly diagnosed as adenocarcinoma) and the posterior mandible was the most affected site. Overall, metastasis as the first indication of malignancy was observed in 125 cases (27.6%), although organ-related differences are missing. The most recent review has been reported by Kirschnick LB et al. in 2020 [37]; although the authors systematically reviewed the literature and used rigid criteria to select papers (from a total of 3346 records collected from the screening of all available database, only 217 studies were selected and 348 cases in total were discussed), clinical-epidemiological data overlap with previous studies (the mandible remains the most affected site and LC the most frequent metastatic tumor in men); in addition, no case with dental implant involvement was included. Importantly, 30% of metastases (87 cases) were discovered before the primary tumor (although the distinction by organ of origin is lacking), and the overall survival rate was reported to be 17.7% and 7.3%, at three and five years, respectively. To date, only single reports or case series have been further added to the previously published and reviewed studies [53,54].

### 4.3. Diagnostic Issues in Metastatic Lung Cancer to Head and Neck

It must always be remembered that, although LC is the most common malignancy worldwide, the lungs are also frequent sites for metastases, and this should always be considered in the differential diagnosis. Additionally, LC definitive diagnosis is often challenging for several reasons; firstly, biopsy samples are usually obtained by bronchoscopy, a percutaneous route, pleural effusions, or from metastatic lesions; therefore, they are frequently small in size and may represent a challenge for pathologists to properly diagnose and classify the cancer from such small samples. The use of immunohistochemistry (IHC) is mandatory for an accurate histological categorization, which remains the basis for deciding further molecular tests for treatment selection in LC patients, but also in differentiating primary LC from metastases [55]. Although there is not a unique marker which can be considered specific for the pulmonary origin, distinct combinations of antibodies may facilitate the identification of the lung as the site of the primary tumor. Among these, TTF-1 is a pneumocyte marker with high sensitivity and specificity for primary lung adenocarcinomas [55,56,57,58,59,60,61,62]; TTF-1 is not expressed in every type of lung adenocarcinoma (Yoshimura) and, moreover, can also be positive in carcinomas from other sites, particularly depending on the antibody clone.

The combination of CK7 with CK20 and CDX2 is usually helpful to differentiate primary lung adenocarcinoma, classically positive for CK7, from metastatic colorectal carcinoma, which is usually negative for CK7 and positive for both CK20 and CDX2 [63,64]. An exception to this immunohistochemical pattern is represented by the enteric-type lung adenocarcinoma, which may have the same immunohistochemical profile of colorectal carcinomas, showing positivity for CK20 and CDX2. Furthermore, it is important to highlight that primary adenocarcinomas from the upper digestive tract can be positive for both CK7 and CK20 and it should not be forgotten that primary lung mucinous adenocarcinomas are also CDX2-positive. The immunohistochemical profile of some primary LCs (mucinous and enteric-type lung adenocarcinoma) may be similar to digestive tract and pancreato-biliary adenocarcinomas, as they are often positive for CK7 and/or CK20 and CDX2 and can be negative for TTF-1 [63,64]. Weak and/or focal positivity for TTF-1 can be observed in poorly differentiated LCs, but usually show a limited value in differentiating pulmonary from extra-pulmonary carcinomas [55,56,57,58,59,60,61]. The use of PAX8, PSA, GATA-3 and estrogen receptors may be helpful in the differential diagnosis, because lung adenocarcinomas essentially show negative nuclear staining for these markers, excluding the possibility of metastatic disease from a carcinoma of Müllerian (PAX8), prostatic (PSA), urothelial (GATA-3), or mammary (GATA-3 and estrogen receptors) origin.

### 4.4. Final Considerations and Discussion about the Herein Reported Cases

In 30% of cases, oral metastasis represents the first manifestation of the cancer [2,5,6,7] and usually indicates an advanced, multiple-metastatic cancer. Hirshberg et al., in one of the first reviews regarding 157 metastases to the oral mucosa in men, reported that the primary tumor was in the lung in 36% of cases, the kidney in 16% and the skin in 15%. In female patients, oral metastases most commonly originate from breast tumors (24%), followed by gynecological (17%), lung (12%), bone (10.3%) and renal tumors (10.3%) [6].

In the current study, seven metastases involved submandibular lymph nodes, thus confirming them as an elective target for lymphogenous metastatic LCs. Their clinical identification is very difficult to achieve, as the differential diagnosis includes inflammatory diseases of the major salivary glands and lymph nodes, equally characterized by a similar unspecific clinical presentation, with painless/painful rapid swelling of a variable consistence too; moreover, benign and malignant primary tumors are certainly to be considered in the diagnostic work-up [2,7,8,9].

In our study, nine cases showed gingival localization (with or without alveolar bone involvement), further demonstrating that periodontal tissue may represent an adjunctive elective target for hematogenous metastatic LCs. Additionally, in almost all the aforementioned cases, a condition promoting periodontal inflammation, such as necrotic tooth roots, broken teeth, peri-implantitis/peri-mucositis and poor dental hygiene, has been constantly observed [10,65,66]. This is in strict accordance with literature data on the predilection for specific sites in the oral cavity of metastatic neoplasms, which may be influenced by peculiar clinical conditions, such as the gingival-periodontal soft tissues of dentates with inflammatory lesions, or the same tissues in edentulous individuals bearing prostheses [2,5,6,7,10,64,65,66,67]. In fact, it has been postulated that in such instances, the re-organization of the local blood flow, following inflammation or induced by the prosthesis pressure, may facilitate the metastatic implantation and growth [2,5,6,7,66,67]. Moreover, jawbones and especially the molar and premolar regions are frequently involved in the metastatic process due to their rich vascularization and high bone marrow content, and metastases may also develop at post-extraction sites, possibly as a consequence of the increased blood flow after blood cloth organization [10].

Unquestionably, gingival metastases from LC preferentially occur in advanced stages, both when they represent the first manifestation of an occult primary tumor or when they are recognized in the clinical work-up of an already known metastatic disease. Currently, the prognosis of lung cancer patients with gingival metastasis is very poor, as the median survival time has been reported to be approximately four months [66] and additionally their treatment with systemic chemotherapy or radiotherapy is not particularly effective. However, surgical excision for bioptic purposes and/or surgical mass reduction in the oral cavity are usually suggested, because may they provide rapid relief of symptoms and may improve dental function and speaking, frequently limited by the rapid growth process and large clinical dimensions [67].

The histological detection of adenocarcinoma in bioptic or fine needle aspiration samples from head and neck neoplasms should always lead to the consideration of a possible secondary localization of a known or unknown neoplasm in the differential diagnosis. Similar cases always represent a challenging situation for pathologists, both to recognize the lesion as a metastatic neoplasm and, additionally, to ascertain the site of the primary tumor. Diagnosing LC, also on primary tumor samples, often requires immunohistochemical evaluation for a correct diagnosis. This is clearly more evident in the case of metastases because the differential diagnosis may be more difficult and may include several neoplastic entities. Thus, the use of immunohistochemistry is strongly recommended to avoid possible pitfalls and to obtain a correct diagnosis, hence allowing an appropriate prosecution of the clinical work-up for patients with advanced neoplastic disease.

In fact, in general months elapsed between the first clinical appearance and the final diagnosis of metastasis; this surely represents a delay in the diagnosis of malignancy, although it is essentially related to the rarity of metastases in the oral cavity and the unspecific and highly variable clinical manifestations; in addition, diagnosis could be further delayed in patients with unknown risk factors (primary malignancy not yet diagnosed), as for eight out of twenty-one of the herein reported cases. In addition, the poor attitude of general dentists to collecting samples for cyto-histological examination of suspicious lesions surely represents an adjunctive contributing factor to such delays. Data from literature generally confirmed a constant delay (about six months) in oral cavity metastasis recognition and diagnosis, thus leading to an overall poor prognosis in such patients [4,5,6,7,8,35,36,37,38,52,53,68,69]. Nevertheless, it is difficult to assess whether overall survival could improve with an earlier onset of therapy as oral metastases are usually recognized in the advanced stage of the disease; however, this should represent a future perspective, especially thanks to the continuous innovation in medical treatments (targeted therapy and immunotherapy) of the past decade possibly associated with conventional chemo-radiotherapy [70,71,72].

## Figures and Tables

**Table 1:** Clinical data of patients.

Case	Age	Sex	Site/Sites	Primary Lung Tumor	Other Sites	First Sign of Disease
1	43	M	Submandibular lymph nodes	Non small cell carcinoma	n.a.	Yes
2	52	M	Submandibular lymph nodes	Non small cell carcinoma	n.a.	No
3	65	M	Gingiva	Non small cell carcinoma	n.a.	No
4	82	F	Gingiva	Squamous carcinoma	n.a.	No
5	57	M	Mandible	Non small cell carcinoma	n.a.	No
6	68	M	Submandibular lymph nodes	Non small cell carcinoma	n.a.	No
7	62	M	Gingiva	Non small cell carcinoma	n.a.	No
8	67	F	Submandibular lymph nodes	Non small cell carcinoma	n.a.	No
9	50	M	Submandibular lymph nodes	Squamous carcinoma	n.a.	Yes
10	79	M	Gingiva/palate	Non small cell carcinoma	n.a.	Yes
11	65	M	Gingiva/tongue	Squamous carcinoma	n.a.	No
12	57	M	Submandibular lymph nodes	Small cell carcinoma	n.a.	No
13	65	M	Submandibular lymph nodes	Squamous carcinoma	n.a.	No
14	80	M	Mandible	Squamous carcinoma	Vertebra	No
15	67	M	Maxillary gingiva	Squamous carcinoma	Vertebra	No
16	65	M	Parotid gland	Small cell carcinoma	n.a.	Yes
17	70	F	Maxillary gingiva Around dental implant	Small cell carcinoma	n.a.	Yes
18	62	M	Maxilla gingiva Around dental implant	Adenocarcinoma	n.a.	Yes
19	65	F	Tongue	Small cell carcinoma	n.a.	Yes
20	72	F	Maxillary gingiva	Adenocarcinoma	n.a.	Yes
21	66	M	Tongue	Small cell carcinoma	Brain	No

**Table 2:** The immunohistochemical panel used for differential diagnosis.

Cases	DIAGNOSIS	CK5/6	CK8/18	CK7	CK20	p40	p63	TTF-1	CDX2	Chromogranin A	Synaptophysin	GATA-3	Estrogen Receptors	PAX 8	PSA
1	Non small cell carcinoma—unclassifiable (undifferentiated carcinoma)	−	+/−	−	−	−	+/−	+/−	−	−	−	−	−	−	−
2	Non small cell carcinoma—unclassifiable (undifferentiated carcinoma)	−/+	+/−	−	−	−	−	+/−	−	−	−	−	−	−	−
3	Non small cell carcinoma—unclassifiable (undifferentiated carcinoma)	−	−/+	−/+	−	−	−/+	+	−	−	−	−	−	−	−
4	Squamous carcinoma	+	−	−	−	+	+	−	−	−	−	−	−	−	−
5	Non small cell carcinoma—unclassifiable (undifferentiated carcinoma)	−	−	−/+	−	−	−	+	−	−	−	−	−	−	−
6	Non small cell carcinoma—unclassifiable (undifferentiated carcinoma)	+/−	−	−	−	−	−/+	+	−	−	−	−	−	−	−
7	Non small cell carcinoma—unclassifiable (undifferentiated carcinoma)	−	+/−	−	−	−	−/+	+/−	−	−	−	−	−	−	−
8	Non small cell carcinoma—unclassifiable (undifferentiated carcinoma)	−/+	+	−	−	−	−	+	−	−	−	−	−	−	−
9	Squamous carcinoma	+	−	−	−	+	+	−	−	−	−	−	−	−	−
10	Non small cell carcinoma—unclassifiable (undifferentiated carcinoma)	−	−/+	+/−	−	−	−	+/−	−	−	−	−	−	−	−
11	Squamous carcinoma	+	−	−	−	+	+	−	−	−	−	−	−	−	−
12	Small cell carcinoma	−	+	−	−	−	−	+	−	+	+	−	−	−	−
13	Squamous carcinoma	+	−	−	−	+	+	−	−	−	−	−	−	−	−
14	Squamous carcinoma	+	−	−	−	+	+	−	−	−	−	−	−	−	−
15	Squamous carcinoma	+	−	−	−	+	+	−	−	−	−	−	−	−	−
16	Small cell carcinoma	−	+	−	−	−	−	+	−	+	+	−	−	−	−
17	Small cell carcinoma	−	+	−	−	−	−	+	−	+	+	−	−	−	−
18	Adenocarcinoma	−	−	+	−	−	−	+	−	−	−	−	−	−	−
19	Small cell carcinoma	−	+	−	−	−	−	+	−	+	+	−	−	−	−
20	Adenocarcinoma, enteric-type	−	−	+	+/−	−	−	+	+/−	−	−	−	−	−	−
21	Small cell carcinoma	−	+	−	−	−	−	+	−	+	+	−	−	−	−

## Data Availability

Not applicable.
